# Pediatric Sjögren Disease: Clinical Features, Diagnostic Challenges, and Outcomes in a Single-Centre Romanian Case Series

**DOI:** 10.3390/jcm15062199

**Published:** 2026-03-13

**Authors:** Mihaela Sparchez, Ioana Filimon, Mirela Crisan, Lidia Man, Simona Corina Senila, Ionut Iarca, Laura Banias, Andreea Liana Bot (Rachisan)

**Affiliations:** 1 Second Pediatric Discipline, “Iuliu Hatieganu” University of Medicine and Pharmacy, 400177 Cluj-Napoca, Romania; 2 Emergency Clinical Hospital for Children Cluj-Napoca, 400370 Cluj-Napoca, Romania; 3 Department of Pediatrics I, “George Emil Palade” University of Medicine, Pharmacy, Sciences and Technology of Târgu-Mureș, 540142 Târgu Mureș, Romania; 4 Emergency Clinical County Hospital Târgu-Mureș, 540136 Târgu Mureș, Romania; 5 Department of Dermatology, County Emergency Clinical Hospital, “Iuliu Hațieganu” University of Medicine and Pharmacy, 400006 Cluj-Napoca, Romania; 6 Emergency Clinical Hospital for Children, 500063 Brașov, Romania; 7 Pathology Department, George Emil Palade University of Medicine, Pharmacy, Science, and Technology of Târgu-Mureș, 540142 Târgu Mureș, Romania; 8 Pathology Department, Emergency Clinical County Hospital Targu-Mureș, 540136 Târgu Mureș, Romania; 9 Department 2—Medical Sciences, Faculty of Nursing and Health Sciences, University of Medicine and Pharmacy Iuliu Hatieganu, 400177 Cluj-Napoca, Romania

**Keywords:** childhood Sjögren, juvenile Sjögren, recurrent parotitis, salivary gland ultrasound, exocrine gland autoimmunity, case series, literature review

## Abstract

**Background/Objectives**: Childhood-onset Sjögren disease (cSjD) is a rare autoimmune disorder with heterogeneous manifestations and ongoing diagnostic challenges, as there are no validated paediatric criteria. Our study aims to characterise the clinical, laboratory, and imaging features of children diagnosed with cSjD at a single Romanian paediatric rheumatology centre between 2015 and 2025 and contextualise these findings within the most recent literature. **Methods:** A retrospective review of 15 consecutive cSjD patients was conducted, including clinical features, autoantibodies, imaging, biopsy findings, treatment, and outcomes. **Results:** Our cohort showed a significant female predominance (80%) and a broad age range at disease onset (3–15 years). Extraglandular manifestations were more common at presentation than glandular phenotypes (53.3% vs. 40%). Lupus-like extraglandular presentations frequently led to initial misdiagnosis as childhood-onset systemic lupus erythematosus (SLE) in our cohort. Sicca symptoms were present at diagnosis in only 3 of 15 patients (20%) and developed later during follow-up in an additional 4 patients (26.7%). Notably, the cohort included novel findings, such as an unprecedented presentation with acute exudative pericarditis complicated by cardiac tamponade. Anti-SSA antibodies and salivary gland ultrasound abnormalities were highly prevalent (86.7% and 100%, respectively). Anti-SSB antibodies were detected in seven patients (46.7%), with titres showing more variability than those of anti-SSA, ranging from just above the positivity threshold to mildly elevated levels. The association with macro-creatine kinase type I was another distinctive feature of this series. Chronic musculoskeletal pain and dryness were our patients’ most frequently reported symptoms at the last assessment, affecting up to 5/15 (33.3%) in each domain. One patient showed irreversible ocular damage during our study. **Conclusions**: Extraglandular presentations of cSjD are highly heterogeneous and diagnostically challenging, often occurring without glandular symptoms. Lupus-like systemic features—including facial vasculitic purpura, with or without arthralgia, and occasional pericarditis, as observed in our cohort—may contribute to frequent initial diagnostic misattribution to SLE. Early salivary gland ultrasonography, targeted autoantibody testing, and selective biopsy are essential for timely diagnosis, underscoring the urgent need for paediatric-specific validated classification criteria.

## 1. Introduction

Childhood-onset Sjögren’s disease (cSjD) is a rare autoimmune exocrinopathy, comprising about 1% of all Sjögren’s disease cases [1]. Compared to adults, children present with a distinct clinical profile, characterised by more frequent recurrent parotid gland enlargement and systemic symptoms, and fewer sicca symptoms at disease onset [2,3,4,5,6,7,8]. Disease can start as early as 4 years old, although most patients are diagnosed between 5 and 13 years [9,10]. Although recent multicentre studies have significantly broadened the current understanding, cSjD remains underrecognised, as its clinical presentation is highly variable [2,3,4,5,6,7,8].

Adult classification criteria, including the 2016 ACR/EULAR criteria [11], are inadequate for diagnosing SjD in children [3,8,12]. As a result, paediatric-specific diagnostic approaches have been proposed, culminating in a recently published clinical algorithm tailored to cSjD [13,14,15]. Objective investigations—such as serology, minor salivary gland biopsy (MSGB), and ocular or salivary functional tests—are used when feasible but lack validated reference standards for this age group, diagnosis often relies on expert clinical judgment [8,15]. The proposed algorithm emphasises the early use of non-invasive salivary gland ultrasonography (SGUS), which has emerged as a valuable diagnostic and monitoring tool in paediatric disease [15]. Given its invasive nature, MSGB is not routinely indicated; however, it should be considered in cases with atypical or concerning features [15].

The British Society of Rheumatology recently published the first guideline for the management of SjD, including children; however, evidence in children and adolescents remains limited, and most conventional immunosuppressive drugs are used off-label in children [16]. Long-term care focuses on monitoring systemic complications, preventing irreversible damage, and preserving quality of life through multidisciplinary follow-up [3,8,15].

This study aims to describe a single-centre paediatric cohort with cSjD, highlighting the heterogeneity of clinical presentation, diagnostic challenges, and therapeutic strategies, and to contextualise our findings within the existing paediatric literature.

## 2. Materials and Methods

### 2.1. Study Design

A retrospective observational study was conducted at the Paediatric Rheumatology Division of the Emergency Clinical Hospital for Children Cluj-Napoca, Romania, a tertiary referral centre. Eligible participants were all consecutive patients diagnosed with cSjD between 2015 and 2025, based on a combined expert opinion that considered clinical evaluation supported by autoantibodies, glandular imaging, or MSGB. Patients with other rheumatic diseases accompanied by potential cSjD overlap were excluded.

We also assessed all newly referred patients during the studied period with suspected cSjD. For those in whom the diagnosis was not confirmed, we reviewed and classified their characteristics and final diagnoses.

Upon admission to our clinic for routine clinical appointments, written informed consent was obtained from parents to use anonymised clinical data.

### 2.2. Data Collection

For the cSjD patients included in the study, the following data were collected from electronic medical records: demographics; presenting symptoms, signs, and additional disease manifestations developed since presentation to the last assessment, defined as per ESSDAI glossary [17]; laboratory results (ANA, SSA, SSB, RF, IgG, C3, C4, FBC, urinalysis); SGUS and/or magnetic resonance imaging (MRI); ophthalmological evaluation; MSGB analysis; additional autoimmune conditions; and treatment strategies ever used for cSjD and follow-up outcomes. We evaluated patient-reported outcomes, disease activity, and damage at the last study assessment using validated adult instruments: the EULAR Sjögren’s Syndrome Patient-Reported Index (ESSPRI) [18], the EULAR Sjögren’s Syndrome Disease Activity Index (ESSDAI) [19], and the Sjögren’s Syndrome Disease Damage Index (SSDDI) [20].

### 2.3. Evaluation of Sicca Syndrome

For ocular dryness, objective signs were documented by an experienced ophthalmologist and deemed sufficient. When possible, a Schirmer test was performed to corroborate the clinical findings.

Oral dryness was evaluated based on subjective symptoms, objective evidence of tongue or gingival mucosal changes consistent with hyposalivation as assessed by an experienced oral medicine specialist, or the presence of recurrent dental caries suggestive of salivary dysfunction.

### 2.4. Ultrasound Evaluation

An experienced ultrasonographer examined the major salivary glands (parotid, submandibular, and, when indicated, lacrimal) using a high-resolution linear transducer (9–15 MHz) to identify abnormal gland architecture at the initial clinic visit and during follow-up assessments as needed. An abnormal SGUS consistent with SjD, such as an inhomogeneous echotexture ranging from a finely granular pattern to multiple hypoechoic areas, with irregular glandular contours, increased parenchymal Doppler flow, and possible hyperechoic fibrotic bands, was considered positive, as SGUS interpretation in children has not been established [15].

For this study, we applied the semiquantitative OMERACT scoring system [21] and reanalysed archived ultrasound images or video clips from the first presentation when available. Accordingly, the scores were defined as follows: score 0, normal parenchyma; score 1, mild inhomogeneity without anechoic or hypoechoic areas and hyperechogenic bands; score 2, moderate inhomogeneity with focal anechoic or hypoechoic areas; and score 3, severe inhomogeneity with diffuse anechoic or hypoechoic areas occupying the entire gland or a fibrous gland [21].

### 2.5. Histological Evaluation

The MSGB was considered confirmatory for cSjD in the presence of focal lymphocytic sialadenitis (focus score ≥ 1 per 4 mm^2^) [11].

## 3. Results

### 3.1. Patient Characteristics

A total of 15 children were identified, 12 (80%) were female, with a median age at symptom onset of 9 years (range 3–15) and a median follow-up of 24 months (range 2 to 56 months) (Table 1). The entire cohort consisted of individuals of White Caucasian ancestry. One patient had a maternal history of SjD, and three additional patients reported a family history of other autoimmune disorders. Thirteen of the 15 individuals ([86.7%]) were diagnosed between 2022 and 2025. Two of them had positive serology for coeliac disease (patients 8 and 13).

### 3.2. Clinical Presentation at Disease Onset

**Figure 1:**
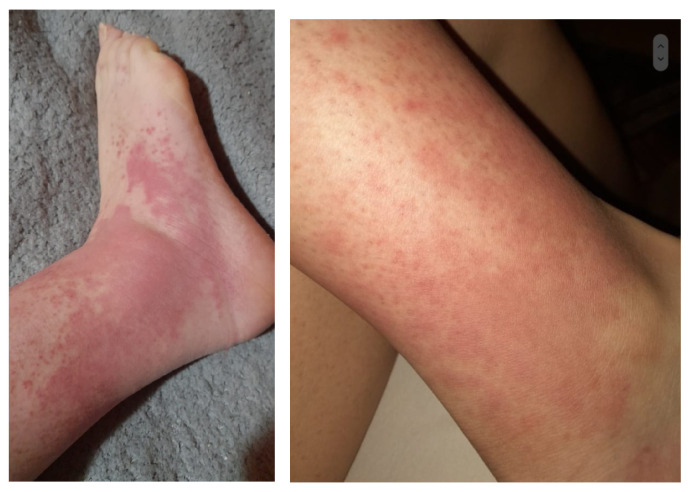
Cutaneous vasculitis in patient 1 distributed symmetrically over the lower legs.

The most common initial manifestations were extraglandular features, observed in eight patients (53.3%). These included persistent or recurrent facial rash in 5 of 15 patients (33%), arthralgia or arthritis in 3 of 15 (20%), recurrent self-limited episodes of cutaneous vasculitis affecting the lower legs with a typical duration of 3–4 days in 1 of 15 (6.7%) (Figure 1), and recurrent pericarditis in 1 of 15 (6.7%) (Table 1).

Glandular manifestations were present before or at initial presentation in six patients (40%), comprising recurrent parotitis in five patients (33%) and lacrimal gland swelling in one patient (6.7%). Sicca symptoms were observed at diagnosis in 3 of 15 patients (20%), with 1 patient experiencing a prolonged disease course of 7 years before diagnosis. Additionally, oral and/or ocular dryness developed during follow-up in four patients (patients 1, 4, 9, and 10).

Fatigue was reported as an accompanying symptom at presentation in 3 of 15 patients (20%), while the Raynaud phenomenon was documented in 1 patient (6.7%).

### 3.3. Laboratory Findings

Antinuclear antibodies (ANA ≥ 1:320 by indirect immunofluorescence) were detected in all our patients with cSjD, except for two who had biopsy-confirmed SjD (patients 10, 15). Of the 15 patients, anti-Ro/SSA antibodies were found in 13 (86.7%), SSB antibodies in 7 (46.7%), and rheumatoid factor (RF) positivity in 13 (86.7%). Anti-SSB positivity was observed in seven patients (46.7%), with antibody levels showing greater variation than anti-SSA, ranging from just above the positivity threshold to mildly elevated titres. Notably, patient 7 initially tested negative for anti-SSB (La) antibodies but later showed mild positivity at 33 U/mL (laboratory cutoff < 15 U/mL). Importantly, all ANA-positive patients exhibited significantly elevated anti-SSA antibody levels (>200 U/mL; laboratory reference < 15 U/mL). In all 13 patients, RF levels were markedly elevated at presentation, ranging from 3- to 22-fold above the upper limit of normal.

All patients had serum IgG concentrations above the age-adjusted laboratory reference ranges (upper limit 14.4–16.7 g/L) at initial evaluation and throughout follow-up. Moreover, five patients (33.3%) displayed markedly elevated levels exceeding 20 g/L.

C3 complement levels remained within normal laboratory reference ranges in all patients throughout the entire follow-up period. In patient 1, C4 was significantly decreased at diagnosis (3.8 mg/dL; normal 10–40 mg/dL), with negative cryoglobulins and normal C3 levels. C4 values gradually normalised over the subsequent five months of therapy.

Cryoglobulinaemia was negative in all patients who were tested (8/15).

Lymphopenia was a common finding in this cohort, present in all patients either at diagnosis or emerging during the course of the disease and remained persistent in some cases.

Patient 6 had persistently elevated creatine kinase levels (between 400 and 650 U/L) without clinical signs of myopathy and with no evidence of muscle inflammation on MRI. She was subsequently diagnosed with type I macro-CK syndrome after identifying a high macro-CK complex (3.2%) through CK isoenzyme electrophoresis.

Specifically, patient 8, who presented with the unusual initial manifestation of pericarditis, demonstrated a serological profile highly suggestive of SjD, including a high-titre ANA (1:640), positivity for anti-SSA, anti-SSB, and anti-Ro52 antibodies, elevated RF (173 IU/mL), and increased serum IgG levels (2253 mg/dL), with negative anti-dsDNA and anti-Sm antibodies.

### 3.4. Salivary Gland Imaging

All individuals showed SGUS findings (parotid, submandibular or lacrimal) consistent with cSjD, which supported and ultimately confirmed the diagnosis based on expert clinical judgement (100%).

**Figure 2:**
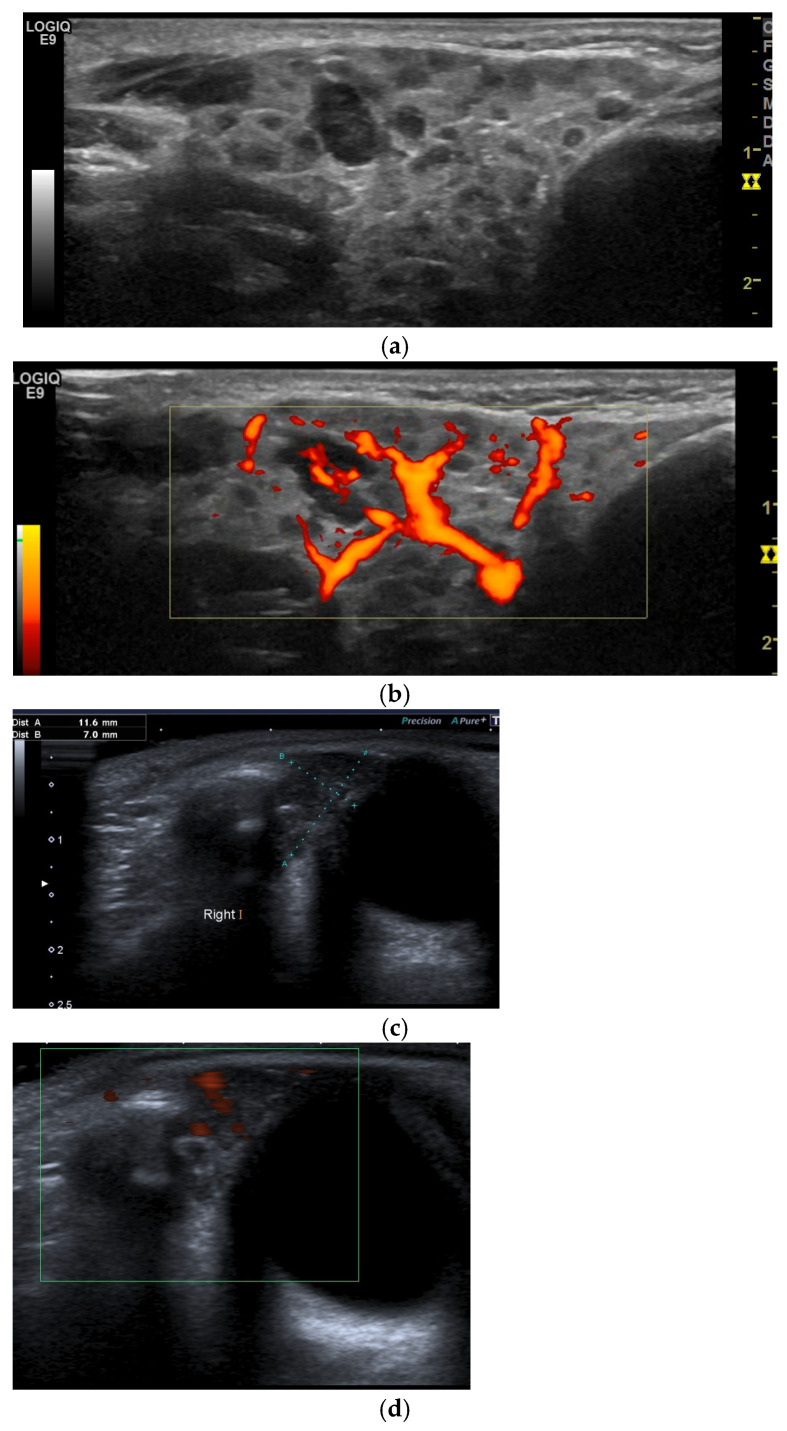
Ultrasound findings of the salivary and lacrimal glands in patient 1. Parotid gland ultrasound in B-mode (**a**) and colour Doppler (**b**) demonstrates an inhomogeneous echotexture with multiple hypoechoic areas, intraglandular lymph nodes, and increased parenchymal vascularity. Lacrimal gland assessment in B-mode (**c**) and Doppler (**d**) shows a normal ultrasonographic appearance.

For 11 (73.3%) patients, archived ultrasound images or video clips were available for re-evaluation using the new OMERACT semiquantitative scoring system (Figure 2). At diagnosis, one patient (6.7%) had an OMERACT score of 1, seven (46.7%) had a score of 2, and three (20%) had a score of 3.

Lacrimal gland ultrasound (LGUS) was performed at initial presentation or during follow-up, and all but one showed no abnormalities in echotexture, vascularity, or gland size, despite significant changes on parotid and submandibular gland assessment.

On the contrary, patient 12, who presented with left lacrimal gland swelling and ocular dryness, had US and MRI abnormalities of the lacrimal glands (increased left-sided gland volume compared to the contralateral side, hypervascularity, and a heterogeneous echotexture). Notably, her SGUS examination was normal. In this patient, a lacrimal gland biopsy and the presence of specific autoantibodies confirmed the diagnosis.

**Figure 3:**
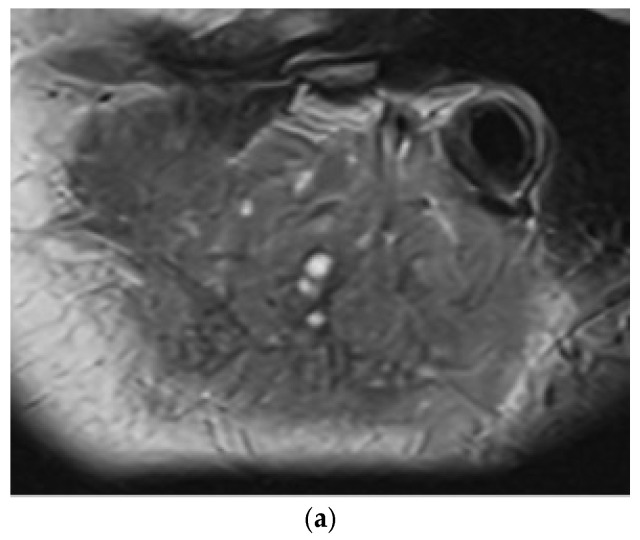

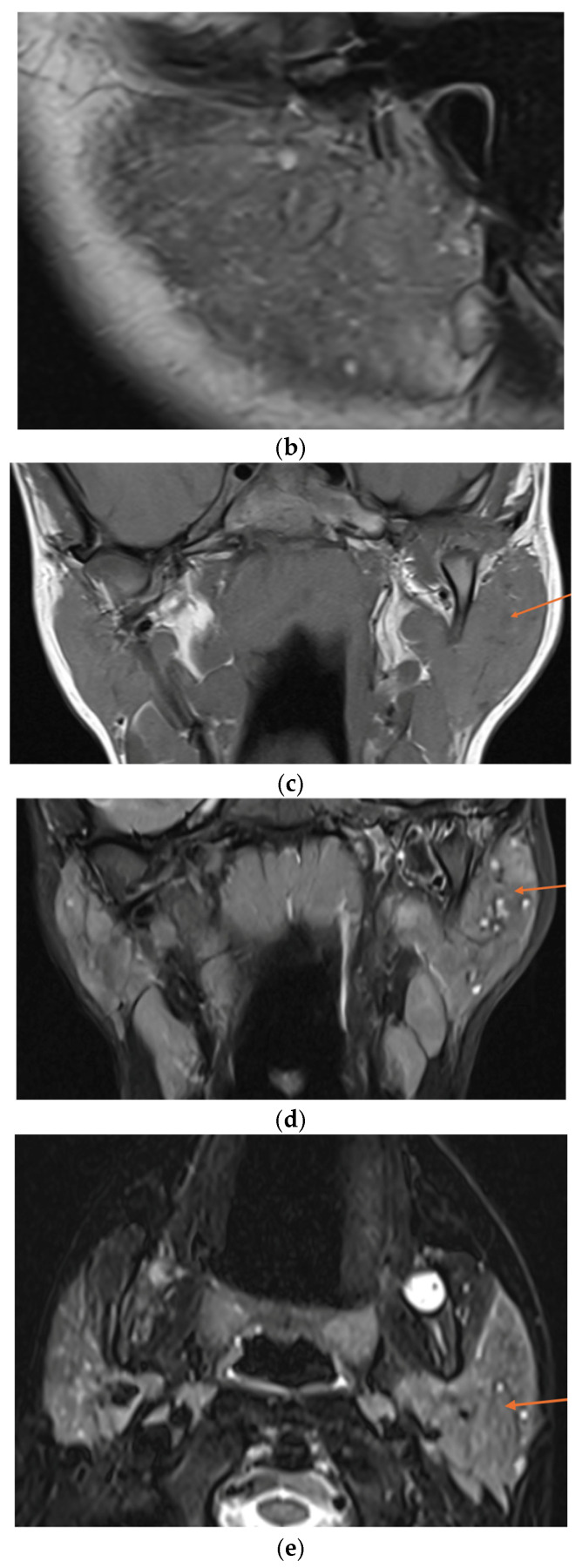
Cervical MRI in patient 15. There is enlargement of both parotid glands, more pronounced on the left. The glands measure 47 × 20 × 11 mm (CC/LL/AP) on the left and 46 × 16 × 10 mm (CC/AP/LL) on the right. Both glands show a heterogeneous structure with T2-hyperintense fluid-like components, cystic in nature, measuring less than 1 cm. Imaging includes sagittal T2 (left and right sides) (**a**,**b**), coronal T1 (**c**), STIR (**d**), and axial STIR (**e**) sequences. The arrows indicate the left parotid gland.

MRI was performed at diagnosis in only three patients with atypical presentations: patient 10, who had persistent submandibular gland enlargement and negative ANA; patient 12, with lacrimal gland swelling and a normal ultrasound appearance of the parotids and submandibular glands; and patient 15, who had a younger age of disease onset and negative ANA (Figure 3). No MRI showed punctate sialectasis or a “salt-and-pepper” appearance. Furthermore, due to persistently elevated creatine kinase levels, patient 6 underwent an MRI to evaluate possible muscular involvement.

### 3.5. Histopathological Findings

An MSGB was recommended for 6 of the 15 children in our cohort (40%), and 4 underwent the procedure. The remaining two had postponed the biopsy at the time this report was prepared. In these cases, the attending physician requested a biopsy when atypical features were present, autoantibodies were negative, ultrasound examination of the gland did not reveal characteristic findings, or diagnostic confirmation was required for any reason.

For the other nine patients (60%), whose clinical presentation, serology, and ultrasound findings were already supportive of the diagnosis, biopsy was considered to have no added value and was not pursued.

**Figure 4:**
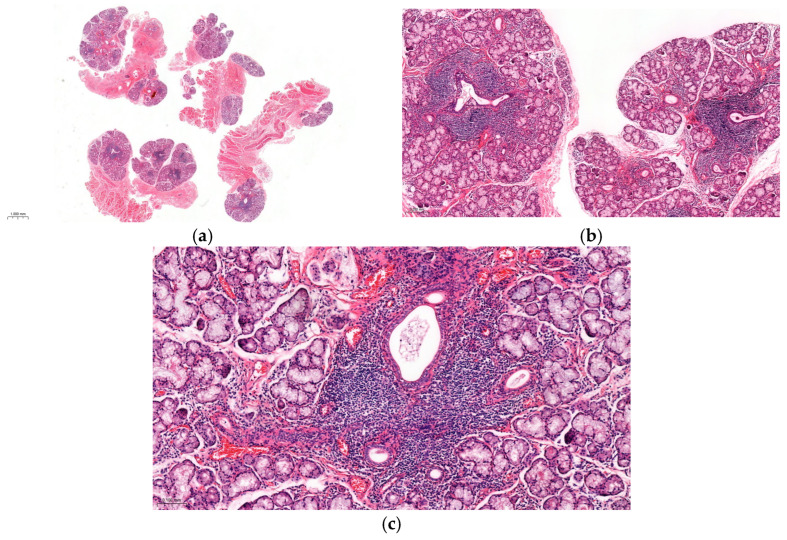
Minor salivary gland histopathology demonstrating focal lymphocytic sialadenitis on H&E staining in patient 7. (**a**) Multiple lymphocytic inflammatory foci (>50 lymphocytes per Focus) (×0.8). (**b**) Periductal inflammatory foci with more than 50 lymphocytes (×5).; (**c**) Periductal focus with more than 50 lymphocytes (×10).

Among the four patients who underwent MSGB or lacrimal gland biopsy (LSGB), three exhibited diagnostic histopathology (focus score ≥ 1), while the other showed dense lymphocytic infiltration but with a focus score < 1. In patient 7, the initial MSGB was non-diagnostic, displaying a diffuse lymphocytic infiltrate. A diagnostic specimen obtained from the repeat biopsy demonstrated focal lymphocytic sialadenitis (Chisholm–Mason grade 3–4) with multiple focal aggregates (>50 lymphocytes per focus) (Figure 4). The girl (patient 12) who presented with lacrimal enlargement underwent an LSGB, which showed lymphocytic dacryoadenitis, consistent with the histopathologic features of Sjögren’s disease. The specimen revealed no granulomas, necrosis, vasculitis, lupus- or MALT-type changes, or IgG4-related features, thereby supporting the diagnosis of cSjD.

In patient 1, who experienced lower-leg vasculitis (Figure 1) characterised by burning sensations and oedema, investigations were undertaken to clarify the underlying mechanism. Laboratory evaluation revealed hypergammaglobulinaemia (elevated IgG, 3493 mg/dL, IgM 300 mg/dL), high rheumatoid factor activity (423 IU/mL), C4 hypocomplementaemia, positive ANA, and anti-Ro/SSA and anti-La/SSB autoantibodies, but without detectable cryoglobulins in serum. Histopathology from a skin biopsy was compatible with hypergammaglobulinaemic vasculitis.

### 3.6. Diagnostic Misclassifications Before cSjD

**Figure 5:**
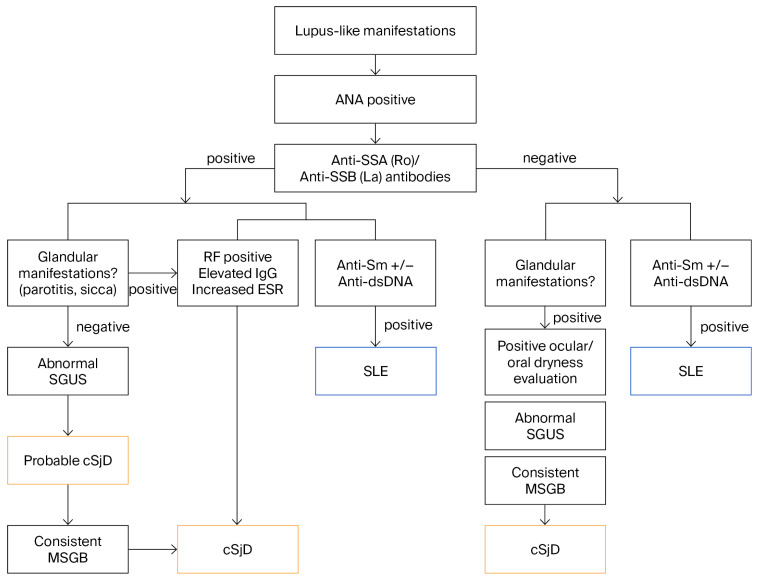
Proposed diagnostic algorithm in patients presenting with lupus-like manifestations. ANA, Antinuclear antibodies; Anti-dsDNA, anti-double-stranded DNA antibodies; ESR, erythrocyte sedimentation rate; MSGB, minor salivary gland biopsy; RF, rheumatoid factor; SGUS, salivary gland ultrasound; Sicca, symptomatic ocular and/or oral dryness; SLE, systemic lupus erythematosus.

Prior diagnostic misattributions before the definitive diagnosis of cSjD included childhood-onset systemic lupus erythematosus (SLE) in five patients (patients 2, 5, 6, 8, and 14; 33.3%). The differential diagnosis with SLE was based on clinical manifestations, detailed immunological and serological profiles, and organ involvement, further supported by characteristic exocrine glandular findings (Figure 5). Additionally, patient 1 was initially diagnosed with systemic vasculitis, while two other patients were misdiagnosed with juvenile idiopathic arthritis.

### 3.7. Treatment and Outcomes

#### 3.7.1. Therapeutic Interventions Initiated at Diagnosis

Eight (53.3%) patients were started on hydroxychloroquine (HCQ) at diagnosis, based on the clinician’s judgement, primarily for systemic manifestations of cSjD and serological activity (notably significant hypergammaglobulinaemia and high rheumatoid factor titres), as well as for glandular involvement. Several patients (46.7%) required more than one therapeutic trial of HCQ for 6–12 months by the time of the final assessment. A single 6-month course of HCQ was sufficient to achieve remission of disease manifestations in three patients (20%).

A short course of non-steroidal anti-inflammatory drugs (NSAIDs) (3–5 days) was used for acute episodes of parotitis. In our cohort, two patients used only NSAIDs during the follow-up period.

Systemic corticosteroids were used in three patients to manage acute episodes of parotitis in selected situations, specifically in the presence of severe pain or prolonged flares that did not respond to NSAIDs (patients 3, 6, 7). Oral prednisone was also used in patient 8 for a severe episode of pericarditis with cardiac tamponade, and in patients 10 and 12, where treatment had been initiated before their presentation to our service by the paediatrician because of marked glandular manifestations.

Conventional synthetic DMARDs, such as azathioprine (AZA), were used in five patients—typically in combination with corticosteroids—as a steroid-sparing strategy.

In addition, colchicine was initiated in patient 8 for pericarditis, alongside systemic corticosteroids, and was continued through the last study assessment.

For dry eye symptoms, all patients received lubricating eye drops at diagnosis and subsequently received short courses of topical corticosteroids under ophthalmologic supervision during episodes of significant keratoconjunctival inflammation. Inflammatory skin rashes were managed with topical corticosteroids for the shortest duration necessary.

In patients with xerostomia, recommendations included the use of artificial saliva, routine brushing with fluoride toothpaste, preventive dental care, and xylitol-containing products to minimise dental caries.

#### 3.7.2. Cumulative Treatment up to the Final Follow-Up

Overall, at the time of the last follow-up, seven of the 15 patients (46.7%) had received short courses of prednisone; 8 (53.3%) had been treated with HCQ, 5 (33.3%) with AZA, 1 (6.7%) with mycophenolate mofetil (MMF), and 1 (6.7%) with Colchicine. None received Rituximab or any biologic drug.

MMF at 900–1000 mg/m^2^ per day, divided into two doses, was started to treat hypergammaglobulinemic vasculitis in patient 1 after an inadequate response to HCQ combined with AZA. This therapy successfully controlled the patient’s extraglandular cutaneous manifestations, lowered serum IgG levels, and normalised complement concentrations.

To facilitate clinical application, we propose a table summarising practical treatment considerations for cSjD, based on currently available expert guidelines and our group’s clinical experience with paediatric patients (Table 2).

#### 3.7.3. Disease Complications During Follow-Up and Outcomes at the Last Assessment

Disease duration at the last assessment ranged from 2 to 56 months (median 24; IQR 6–38). At the final follow-up, the median ESSPRI score was 0 (range 0–2.67; IQR 0–1.33), and the median ESSDAI score was 1 (range 0–8; IQR 0–1), indicating mild subjective symptom burden and low disease activity, with residual activity mainly seen in two patients in the glandular, articular, or biological domains.

Chronic musculoskeletal pain and dryness have been most frequently reported by our patients at the last assessment, affecting up to 5/15 (33.3%) in each of these ESSPRI domains. Dryness was clinically significant in only one patient, with a score of 5/10 on a visual analogue scale. Fatigue was reported by three patients at the last assessment, with significant fatigue only in patient 9 (5/10 cm on a visual analogue scale).

Only one girl (patient 3) showed irreversible ocular damage during our study, including impaired tear flow, with an SSDSI score of 1, confirmed by an ophthalmology specialist who observed a clear decrease in tear secretion (abnormal Schirmer test). Two cases developed chronic keratoconjunctivitis. No cases of lymphoma or major chronic organ involvement were observed in our cohort during follow-up. Patient 5, who initially presented with proteinuria, was closely monitored by a nephrologist. After the initial episode, no further urinary protein losses or other signs of renal involvement have been documented to date.

Patient 8 experienced a recurrence of pericarditis with cardiac tamponade following an abrupt tapering of corticosteroids and colchicine, and pericardiocentesis was performed. Upon reinitiation of treatment, the pericarditis resolved and has remained controlled to the present time. Other potential causes of pericarditis were excluded in this case.

No abnormalities in serum amylase or lipase levels, nor any structural pancreatic changes, were detected.

Parotitis episodes improved during follow-up in six patients [40%], mainly with treatment involving NSAIDs, HCQ, and/or corticosteroids. In patients who did not exhibit these features at diagnosis, sicca symptoms and recurrent parotitis appeared later in the disease course in four and five cases, respectively.

Lymphopenia was common during the disease course in our patients, with or without systemic medications (70%).

### 3.8. Characteristics and Final Diagnoses of Non-Sjögren Cases

Final diagnoses among patients referred with suspected cSjD most commonly included juvenile recurrent idiopathic parotitis, false-positive SSA or SSB results on qualitative serological assays, chronic fibrosing sialadenitis, idiopathic orbital myositis, autoimmune thyroiditis, and IgG4-related disease.

## 4. Discussion

Our findings are closely in accordance with those reported in recent high-quality paediatric cohorts from various international workgroups, all of which highlight the key clinical features of cSjD [1,2,3,4,5,6,7,8]. Additionally, our cohort offers novel observations, including previously unreported presentations such as pericarditis complicated by cardiac tamponade and co-occurrence with type I macro–creatine kinase syndrome. We also confirm the existence of rare disease phenotypes characterised by isolated lacrimal gland involvement without major salivary gland disease. Furthermore, in our cohort, extraglandular presentation was more common and posed a greater diagnostic challenge. Overall, these findings further emphasise the heterogeneity of the clinical spectrum in children and reinforce the significant diagnostic challenges associated with cSjD.

This single-centre cohort from a 10-year observational study showed a clear female predominance and a wide age range at onset, including very early disease presentation as young as 3 years old, findings consistent with those reported in the literature [1,2,3,9,10]. Notably, nearly all patients in our cohort were diagnosed within the past four years, raising the question of whether the incidence of cSjD has increased in recent years. To date, no published data support a true temporal rise in disease incidence. Although other authors have similarly noted a recent increase in published paediatric case series and cohort studies [15], this trend may more plausibly reflect improved disease recognition, heightened awareness, and increased reporting rather than a genuine increase in occurrence. Nonetheless, delayed diagnosis and under-recognition likely persist, and additional undiagnosed cases may remain.

Recurrent swelling of the parotid gland is a key diagnostic feature of cSjD and is considered one of the most common manifestations at onset, often preceding sicca symptoms or other systemic signs, and reported in approximately 33–91% of paediatric cases [1,3,5,6,7,8]. Parotitis inversely correlates with age, being less common in adult-onset SjD, which highlights its diagnostic significance in paediatric populations [3]. In our series, recurrent parotitis was present at initial presentation in only five patients (33%), but developed later in another five patients, thus supporting the diagnosis over time. Similarly, sicca symptoms were uncommon early in the disease and appeared after 2–3 years of progression in about one-third of patients.

Children presenting with extraglandular manifestations pose the greatest diagnostic challenge, as their clinical presentation is highly heterogeneous and may be more severe. Recently published diagnostic algorithms for children with cSjD-like presentations indicate that the extraglandular pathway is the least sensitive for establishing a diagnosis of cSjD [15]. Patients in this group often have few or no sicca symptoms and may lack obvious inflammation of the salivary or lacrimal glands. Notably, in our cohort, this type of initial presentation was more frequent than presentations dominated by parotitis (53.3%). Lupus-like clinical features were present at disease onset in seven patients, manifesting as facial vasculitic purpura with or without associated arthralgia; some were initially considered by the general paediatrician to have SLE. The presence of positive ANA (fine-speckled pattern), moderate- to high-titre SSA ± SSB, the absence of specific lupus antibodies (anti-dsDNA, anti-Sm), high titres of RF, and suggestive SGUS features at presentation were key elements in considering cSjD. According to current paediatric diagnostic algorithms [15], these patients meet criteria for probable cSjD, and a minor salivary gland biopsy is recommended to confirm the diagnosis. In our cases, MSGB was not performed as serology and ultrasound were suggestive of cSjD at that time. Moreover, five of them developed typical glandular manifestations during the course of the disease.

Additionally, both SLE and SjD may overlap in paediatric populations and have been previously documented [8,27]. The presence of specific lupus antibodies, hypocomplementemia, pancytopenia, or specific lupus organ manifestations (such as lupus nephritis) should raise awareness of this potential association, as these cases are uncommon and often associated with increased mortality and a poorer prognosis than in adults [28].

One patient in our cohort experienced recurrent episodes of vasculitis in the lower legs during the first four years of the disease as the initial symptom. These episodes occurred without other systemic features or renal involvement and preceded other classic signs of SjD, tending to be more aggressive and persistent over time. This manifestation is recognised in children diagnosed with SjD, most commonly presenting as palpable purpura caused by small vessel leukocytoclastic vasculitis. Differentiating cryoglobulinemic vasculitis from other vasculitis subtypes (e.g., hypergammaglobulinemic or urticarial vasculitis) is important for prognosis and management. In her case, histopathology from a skin biopsy was compatible with hypergammaglobulinemic vasculitis. The patient continued to experience recurrent vasculitic flares despite receiving AZA and prednisone. MMF was therefore introduced. Recently published guidelines for the management of SjD recommend using DMARDs only for selected systemic complications; however, MMF is not specifically endorsed due to limited evidence supporting its use in this context. Furthermore, MMF is not recommended in either the Japanese or North American guidelines [22,23,24,25,26]. In our case, MMF resulted in effective control of cutaneous involvement and was associated with reductions in serum IgG levels and increases in complement concentrations over the course of the disease, consistent with previous reports [29]. MMF may therefore be considered in selected cases, as it is generally well tolerated and associated with fewer and less severe adverse effects compared to Rituximab.

Rare but illustrative presentations, such as reported cases of bilateral ranulas [30,31,32] and pulmonary haemorrhage [33,34], underscore the broad clinical spectrum of cSjD and highlight the need for heightened diagnostic awareness. In our series, one patient presented with symptomatic acute exudative pericarditis as the initial disease manifestation. His immunological evaluation revealed findings strongly supportive of SjD, and bilateral parotid ultrasound abnormalities were consistent with the diagnosis, despite the absence of objective ocular or oral dryness. Application of paediatric diagnostic algorithms via the extraglandular pathway classified the case as probable cSjD [15]. Accordingly, an MSGB was recommended to confirm the diagnosis, as in the other patient with extraglandular presentation in our cohort; however, the family deferred the procedure at the time of reporting. During follow-up, the patient also developed objective parotitis.

To our knowledge, no cases of clinically significant, symptomatic acute exudative pericarditis complicated by cardiac tamponade have been reported in children with cSjD in the paediatric literature to date. A single case of cSjD has been described with an initial presentation of haemolytic uremic syndrome and pericarditis, followed by a fatal course due to pulmonary haemorrhage; however, the constellation of severe associated systemic manifestations in that report may have contributed to the occurrence of pericarditis and limits direct comparison [34]. In contrast, pericardial involvement, although uncommon, has been documented in adult-onset SjD, most frequently presenting as a subclinical pericardial effusion or acute exudative pericarditis, and, more rarely, as chronic constrictive pericarditis [35,36].

The association with macro-creatine kinase was another distinctive feature of our series. Macro-creatine kinase type I, recognised as a benign cause of elevated CK, often seen in autoimmune conditions, especially in females, is most often linked to other autoimmune conditions; however, its co-occurrence with primary SjD has not been documented in published case series or reviews to date [37,38].

Anti-SSA (anti-Ro) and anti-SSB (anti-La) autoantibodies, together with RF, demonstrate high diagnostic specificity for cSjD (94.7%, 96.0%, and 95.7%, respectively), but only moderate sensitivity (55.6%, 14.8%, and 33.3%, respectively) [39]. Both the paediatric diagnostic criteria proposed by Bartůňková et al. [13] and more recent diagnostic algorithms [15] allow either anti-SSA or anti-SSB positivity to fulfil the serological component of diagnosis, given that the true prevalence and diagnostic value of anti-SSB antibodies in children remain uncertain.

In our cohort, anti-SSA antibodies were frequently detected, often at very high titres, with a positivity rate of 86.7%, consistent with findings from major paediatric cohorts [1,3,4,8]. Anti-SSB antibodies were identified in seven patients (46.7%), with titres showing greater variability than those of anti-SSA, ranging from just above the positivity threshold to mildly elevated concentrations. Longitudinal changes in anti-SSB status were observed in one patient, who initially tested negative and subsequently developed low-titre positivity. Importantly, no patient demonstrated isolated anti-SSB positivity in the absence of anti-SSA antibodies. Furthermore, isolated positivity for anti-La/SSB antibodies is not included in the 2016 ACR/EULAR classification criteria, which are currently the most widely used criteria used for SjD [11]. The clinical significance of anti-SSB in cSjD, therefore, remains to be fully elucidated.

Seronegative cSjD is a recognised entity, and diagnosis should not be excluded solely on the basis of negative anti-SSA or anti-SSB serology, as illustrated by two patients in our cohort and supported by recent reports [2,40]. Conversely, the widespread availability of qualitative autoantibody assays, when applied without an appropriate clinical context, has contributed to an increasing number of referrals for suspected autoimmune disease in children lacking compatible clinical features, often driven by heightened parental anxiety. Limiting immunological testing to cases supported by careful clinical assessment may improve diagnostic accuracy and substantially reduce unnecessary investigations and parental distress.

Parotid and submandibular ultrasound has become a commonly used first-line imaging modality for assessing salivary gland inflammation, is inexpensive, easily repeatable, and non-invasive, with additional advantages in paediatric assessment [15,41]. SGUS supported the diagnosis of cSjD in all our patients, based on characteristic features of SjD that were evident from the initial presentation. It is an operator-dependent technique, but standardised protocols such as the OMERACT scoring system [21] can significantly enhance its reliability and reproducibility. Based on our experience, SGUS proved highly informative, particularly in patients with extraglandular manifestations at presentation, and was consistent with the results of other studies [5,6]. In addition to its diagnostic role, SGUS may aid in monitoring for complications, including lymphoma [15,41].

LGUS may be particularly valuable for identifying rare disease phenotypes characterised by isolated lacrimal gland involvement in the absence of major salivary gland disease, as observed in one patient from our cohort. However, because the diagnostic performance of LGUS is generally lower and less standardised than that of SGUS [42,43,44], additional investigations—including MRI and tissue biopsy—were required to confirm the diagnosis of cSjD. Notably, in other patients with ultrasound findings suggestive of SjD affecting the major salivary glands, LGUS revealed no abnormalities of the lacrimal glands.

MSGB is a key diagnostic tool in cSjD, particularly when clinical and serological findings are insufficient or atypical, and it demonstrates good diagnostic sensitivity and specificity [45]. However, given its invasive nature and the lack of normative paediatric histopathological data [15], it is not routinely recommended. Repeat biopsy may be considered when initial results are negative or equivocal and clinical suspicion remains high, as in our patient. Such decisions should be individualised, carefully balancing procedural risk against potential diagnostic benefit, especially in the context of evolving clinical features or serological changes.

## 5. Conclusions

Our findings highlight the marked heterogeneity of childhood-onset SjD, with extraglandular manifestations frequently predominating and often occurring in the absence of sicca or glandular symptoms, thereby posing significant diagnostic challenges. Lupus-like systemic features—including facial vasculitic purpura, with or without arthralgia, and occasional pericarditis, as observed in our cohort—may contribute to frequent initial diagnostic misattribution to SLE. Early use of salivary gland ultrasonography, comprehensive autoantibody profiling, and selective salivary gland biopsy in atypical cases is essential for timely and accurate diagnosis, underscoring the urgent need for paediatric-specific classification criteria.

## Figures and Tables

**Table 1:** Clinical, laboratory, imaging, treatment, and outcomes of our 15 children with cSjD.

Patient	Sex/Age at Onset (yrs)	Age at Diagnosis (yrs)	Presenting Feature	Glandular Features ^1^	Extraglandular Features ^2^	Autoantibodies	SGUS at Diagnosis (OMERACT Grade 0–3)	MSGB	Treatment ^3^	Outcome Follow-Up Duration ESSPRI; ESSDAI; SSDDI at Last Visit
1	F/12	16	Cutaneous vasculitis Fatigue	Sicca Keratoconjunctivitis	Skin vasculitis flares Lymphopenia Intermittent anaemia	ANA (+) SSA (+) SSB (+) RF (+)	SGUS (+) (grade 2)	ND	CS, HCQ, AZA, MMF	Vasculitis flares controlled after MMF initiation; Chronic keratoconjunctivitis; Sicca Fatigue 38 months 1.33; 1; 0
2	F/13	14	Facial rash	Sicca (−) Parotitis	Lymphopenia Fatigue	ANA (+) SSA (+) SSB (+) RF (+)	SGUS (+) (grade 2)	ND	HCQ	Parotitis episodes improved Persistent lymphopenia 22 months 0; 0; 0
3	F/6	13	Raynaud Xerophthalmia Keratoconjunctivitis Fatigue	Sicca (+) Parotitis	Arthritis Raynaud	ANA (+) SSA (+) SSB (−) RF (+)	SGUS (+) (grade 3)	ND	CS, HCQ, AZA	Tear flow impairment Chronic keratoconjunctivitis Sicca 36 months 1; 1; 1
4	F/10	11	Facial rash Arthralgia	Xerophthalmia Acute keratoconjunctivitis	Arthritis Skin rash	ANA (+) SSA (+) SSB (−) RF (+)	SGUS (+) (grade 2)	ND	HCQ AZA	Sicca stable Lymphopenia 28 months 1.33; 1; 0
5	F/4	5	Facial rash	Sicca (−)	Hepatitis Transient proteinuria Skin rash	ANA (+) SSA (+) Ro-52 (+) SSB (−) RF (+)	SGUS (+)	ND *	HCQ	Good response Intermittent lymphopenia 24 months 0; 0; 0
6	F/7	9	Facial rash Arthralgia	Recurrent parotitis	Arthritis Recurrent skin rashes Lymphopenia	ANA (+) SSA (+) SSB (+) RF (+)	SGUS (+) (grade 2)	ND	HCQ Prednisone	Parotitis flares improved initially, but became more frequent after stopping HCQ Fatigue Athralgia 40 months 1.67; 8; 0
7	M/5	7	Recurrent parotitis	Recurrent parotitis Sicca (−)	None	ANA (+) SSA (+) SSB (+) RF (+)	SGUS (+) (grade 3)	FLS (+) FS > 1	HCQ Prednisone	Parotitis- no new flares under HCQ 11 months 0; 1; 0
8	M/14	14	Recurrent pericarditis Fever	Recurrent parotitis	Recurrent pericarditis with one episode of cardiac tamponade	ANA (+) SSA (+) SSB (+) RF (+)	SGUS (+) (grade 2)	ND *	CS, Colchicine	Pericarditis improved with treatment 6 months 0; 1; 0
9	F/15	16	Recurrent parotitis	Xerophthalmia Xerostomia	Poliarthralgia	ANA (+) SSA (+) SSB (−) RF (+)	SGUS (+) (grade 2)	ND	HCQ	Stable sicca Fatigue Arthralgia Parotitis improved 8 months 4; 1; 0
10	F/12	13	Recurrent parotitis	Xerostomia	Arthritis Fatigue	ANA (−) SSA (−) SSB (−) RF (+)	SGUS (+) (grade 3)	FLS (+) FS > 1	AZA, CS	Stable sicca Parotitis improved 56 months 0; 1; 0
11	F/15	15	Poliarthralgia	none	Poliarthralgia	ANA (+) SSA (+) SSB (+) RF (+)	SGUS (+)	ND	NSAID	No new manifestations Chronic arthralgia 6 months 1; 0; 0
12	F/9	9	Ocular dryness Left lacrimal gland swelling Fatigue	Xerophthalmia	None	ANA (+) SSA (+) Ro-52 (+) SSB (−) RF (−) IgG (−)	Parotid and submandibular US score 0 Lacrimal gland hypertrophy and increased parenchymal flow on Doppler	Lacrimal biopsy: DLS	AZA, CS	Good response, no new flares 42 months 0; 0; 0
13	F/7	8	Xerophthalmia Recurrent parotitis	Sicca (+) Glandular swelling Recurrent vaginitis Intermittent blepharitis Xerostomia	Joint and muscular pain	ANA (+) SSA (+) SSB (−) RF (+)	SGUS (+)	ND	MTX	Sicca stable Arthralgia 3 months 2.67; 0; 0
14	F/8	9	Facial rash	Parotitis	Poliartralgia	ANA (+) SSA (+) SSB (+) RF (+)	SGUS (+) (grade 1)	ND	NSAIDs	Sicca (−) 29 months 0; 0; 0
15	M/3	4	Recurrent Parotitis	none	None	ANA (−) SSA (−) SSB (−) RF (−)	SGUS (+) (grade 2)	FLS (+) FS > 1	NSAIDs	Parotitis unresponsive to NSAIDs; 2 months 0; 4; 0

^1^ Glandular symptoms and signs developed since presentation to the last assessment, defined as per ESSDAI glossary [17]; ^2^ Extraglandular features developed since presentation to the last assessment, defined as per ESSDAI glossary [17]; ^3^ Medication ever given for Sjogren disease; ND = not done; (+) = positive/increased levels; (−) = negative/normal levels; SGUS = salivary gland ultrasound, FLS: focal lymphocytic sialadenitis; FS = focus score per 4 mm^2^; DLS: diffuse lymphocytic sialadenitis; HCQ = hydroxychloroquine; CS = corticosteroids; MTX = methotrexate; MMF = mycophenolate mofetil; AZA = azathioprine; ESSPRI = EULAR Sjogren’s Syndrome Patient Reported Index; ESSDAI = EULAR Sjogren’s Syndrome Disease Activity Index; SSDDI = Sjogren’s Syndrome Disease Damage Index; * Biopsy proposed but postponed by the parent.

**Table 2:** Treatment options and practical considerations in cSjD based on current expert guidelines [8,16,22,23,24,25,26] and our group’s clinical experience.

Pharmacological Interventions	Clinical Indications and Practical Considerations
**Local/Topical Therapy**	
Topical artificial tears Vitamin A-containing eye ointments	Dry eye disease
Topical ciclosporin 0.05%, 0.1%	Persistent ocular surface inflammation not adequately controlled with artificial tears monotherapy (under ophthalmic supervision). Notes: No published dosage for topical ciclosporin in children. It can take 4–12 weeks to observe a treatment response.
Topical ophthalmic corticosteroids (low potency)	Ocular surface inflammation/keratitis Notes: Use short courses only (e.g., 2–4 weeks). Ophthalmologic supervision is required. Monitor intraocular pressure.
Saliva substitutes Adjunctive measures: regular dental care; regular brushing with fluoride toothpaste; xylitol-containing products as an alternative to sugar.	Oral dryness
Topical corticosteroids (cutaneous use, low potency)	Mild cutaneous inflammatory lesions Notes: Short treatment courses. Monitor for skin atrophy.
**Systemic Therapy**	
NSAIDs	Parotitis (short treatment course, e.g., 3–5 days) Arthralgia, Arthritis Myalgia
Corticosteroids	Not routinely indicated in cSjD Short courses of oral CS for recurrent or resistant episodes of parotitis. Specific systemic disease manifestations such as lung disease, cytopaenias, or renal involvement. Notes: Use the minimum effective dose and shortest duration necessary to control systemic disease.
cDMARDS	Not recommended for routine use in cSjD unless there is evidence of specific-organ involvement. Off-license use in children with cSjD from 2 years of age, based primarily on evidence derived from adult studies and smaller, low-quality paediatric studies. Some cDMARDs are preferentially used for certain indications.
Hydroxychloroquine	Initial systemic therapy. A trial of HCQ for 6–12 months is recommended by the BSR guideline for cSjD associated with significant fatigue and systemic symptoms. Recurrent/resistant parotitis Significant hypergammaglobulinemia Cutaneous manifestations (e.g., purpura)
Methorexate	Significant inflammatory arthritis
Mycophenolate mofetil	SjD-associated interstitial lung disease SjD-associated agranulocytosis Refractory cutaneous vasculitis Hypergammaglobulinemia
Cyclophosphamide	SjD-associated interstitial lung disease Cryoglobulinemic vasculitis SjD-associated myelopathy Refractory thrombocytopaenia Membranoproliferative glomerulonephritis
Azathioprine	In combination with corticosteroids as steroid-sparing strategy.
Biologic therapies	Biologic therapies may be considered off-label in children with severe and refractory cSjD, based mainly on evidence extrapolated from adult studies and limited paediatric reports. At present, biologic agents are not routinely recommended in SjD, except for the treatment of selected systemic complications.
Rituximab	MALT lymphoma Severe neuropsychiatric manifestations Cryoglobulinemia Cryoglobulinemic vasculitis Severe parotid swelling SjD-associated pulmonary disease SjD-associated peripheral neuropathy
Belimumab	Rescue therapy for patients with severe systemic disease refractory to conventional immunosuppressive therapy and rituximab. Selected cases with recurrent parotid swelling. Use in children from 5 years of age.
Other treatments used off licence in cSjD	
Intravenous immunoglobulins	Not routinely recommended in SjD except for specific systemic complications. Refractory SjD-associated myositis
Colchicine	Hypergammaglobulinaemic purpura Non-cryoglobulinaemic vasculitis Granulomatous panniculitis Pericarditis
Pilocarpine	Severe symptoms of dryness

cDMARDS = conventional disease-modifying anti-rheumatic drugs; CS = corticosteroids; cSjD = childhood-onset Sjogren disease; HCQ = hydroxychloroquine.

## Data Availability

The original contributions presented in this study are included in the article. Further inquiries can be directed to the corresponding author.

## Abbreviations

The following abbreviations are used in this manuscript:

cSjD	Childhood-onset Sjögren’s disease
FBC	Full blood count
ANA	Antinuclear antibody
SSA	Anti-SSA(Ro) antibodies
SSB	Anti-SSB(La) antibodies
RF	Rheumatoid factor
SGUS	Salivary gland ultrasound
MSGB	Minor salivary gland biopsy
IgG	Immunoglobulin G

## References

[1] Ramos-Casals M., Acar-Denizli N., Vissink A., Brito-Zerón P., Li X., Carubbi F., Priori R., Toplak N., Baldini C., Faugier-Fuentes E. (2021). Childhood-Onset of Primary Sjögren′s Syndrome: Phenotypic Characterization at Diagnosis of 158 Children. Rheumatology.

[2] Kılbaş G., Ayduran S., Şener S., Coşkuner T., Ulu K., Kısaoğlu H., Aslan E., Könte E.K., Arslanaoğlu C., Aydin T. (2025). Results of a Nationwide Multicenter Study in Childhood Sjögren Disease. J. Rheumatol..

[3] Basiaga M.L., Stern S.M., Mehta J.J., Edens C., Randell R.L., Pomorska A., Irga-Jaworska N., Ibarra M.F., Bracaglia C., Nicolai R. (2021). Childhood Sjögren Syndrome: Features of an International Cohort and Application of the 2016 ACR/EULAR Classification Criteria. Rheumatology.

[4] Marino A., Romano M., Giani T., Gaggiano C., Costi S., Singh R., Mehta J.J., Liebermaln S.M., Cimaz R. (2021). Childhood Sjogren′s Syndrome: An Italian Case Series and a Literature Review-Based Cohort. Semin. Arthritis Rheum..

[5] Legger G.E., Erdtsieck M.B., de Wolff L., Stel A.J., Los L.I., Verstappen G.M., Spijkervet F.K., Vissink A., van der Vegt B., Kroese F.G. (2021). Differences in Presentation Between Paediatric- and Adult-Onset Primary Sjögren′s Syndrome Patients. Clin. Exp. Rheumatol..

[6] Hammenfors D.S., Valim V., Bica B.E.R.G., Pasoto S.G., Lilleby V., Nieto-González J.C., Silva C.A., Mossel E., Pereira R.M.R., Coelho A. (2020). Juvenile Sjögren′s Syndrome: Clinical Characteristics with Focus on Salivary Gland Ultrasonography. Arthritis Care Res..

[7] Virdee S., Greenan-Barrett J., Ciurtin C. (2017). A Systematic Review of Primary Sjögren′s Syndrome in Male and Paediatric Populations. Clin. Rheumatol..

[8] Ciurtin C., Peng J., Taylor-Gotch R., Peckham H., Wilson R., Al Obaidi M., Jury E.C., PReS Childhood Sjögren′s Interest Group (2025). Clinical phenotypes, classification, and long-term outcomes of childhood-onset Sjögren′s disease into adulthood: A single-centre cohort study. Lancet. Rheumatol..

[9] Cimaz R., Casadei A., Rose C., Bartunkova J., Sediva A., Falcini F., Picco P., Taglietti M., Zulian F., Cate R.T. (2003). Primary Sjögren syndrome in the paediatric age: A multicentre survey. Eur. J. Pediatr..

[10] Ostuni P.A., Ianniello A., Sfriso P., Mazzola G., Andretta M., Gambari P.F. (1996). Juvenile onset of primary Sjögren′s syndrome: Report of 10 cases. Clin. Exp. Rheumatol..

[11] Shiboski C.H., Shiboski S.C., Seror R., Criswell L.A., Labetoulle M., Lietman T.M., Rasmussen A., Scofield H., Vitali C., Bowman S.J. (2017). 2016 American College of Rheumatology/European League Against Rheumatism classification criteria for primary Sjogren′s syndrome: A consensus and data-driven methodology involving three international patient cohorts. Ann. Rheum. Dis..

[12] Yokogawa N., Lieberman S.M., Sherry D.D., Vivino F.B. (2016). Features of childhood Sjogren′s syndrome in comparison to adult Sjogren′s syndrome: Considerations in establishing child-specific diagnostic criteria. Clin. Exp. Rheumatol..

[13] Bartůnková J., Sedivá A., Vencovský J., Tesar V. (1999). Primary Sjögren′s syndrome in children and adolescents: Proposal for diagnostic criteria. Clin. Exp. Rheumatol..

[14] Houghton K., Malleson P., Cabral D., Petty R., Tucker L. (2005). Primary Sjögren′s syndrome in children and adolescents: Are proposed diagnostic criteria applicable?. J. Rheumatol..

[15] Stern S.M., Basiaga M.L., Cha S., Thatayatikom A., Treemacki E.B., Randell R.L., Dizon B.L.P., Appenzeller S., Edens C., Orrock J.E. (2025). Diagnosing a child presenting with symptoms suggesting Sjögren′s disease: A tool for clinical practice. Rheumatology.

[16] Price E.J., Benjamin S., Bombardieri M., Bowman S., Carty S., Ciurtin C., Crampton B., Dawson A., Fisher B.A., Giles I. (2025). British Society for Rheumatology guideline on management of adult and juvenile onset Sjögren disease. Rheumatology.

[17] Seror R., Bowman S.J., Brito-Zeron P., Theander E., Bootsma H., Tzioufas A., Gottenberg J.-E., Ramos-Casals M., Dörner T., Ravaud P. (2015). EULAR Sjögren′s syndrome disease activity index (ESSDAI): A user guide. RMD Open.

[18] Seror R., Ravaud P., Mariette X., Bootsma H., Theander E., Hansen A., Ramos-Casals M., Dörner T., Bombardieri S., Hachulla E. (2011). EULAR Sjogren′s Syndrome Patient Reported Index (ESSPRI): Development of a consensus patient index for primary Sjogren′s syndrome. Ann. Rheum. Dis..

[19] Seror R., Ravaud P., Bowman S.J., Baron G., Tzioufas A., Theander E., Gottenberg J.-E., Bootsma H., Mariette X., Vitali C. (2010). EULAR Sjögren′s Task Force. EULAR Sjogren′s syndrome disease activity index: Development of a consensus systemic disease activity index for primary Sjogren′s syndrome. Ann. Rheum. Dis..

[20] Vitali C., Palombi G., Baldini C., Benucci M., Bombardieri S., Covelli M., Del Papa N., De Vita S., Epis O., Franceschini F. (2007). Sjögren′s Syndrome Disease Damage Index and disease activity index: Scoring systems for the assessment of disease damage and disease activity in Sjögren′s syndrome, derived from an analysis of a cohort of Italian patients. Arthritis Rheum..

[21] Jousse-Joulin S., D′Agostino M.A., Nicolas C., Naredo E., Ohrndorf S., Backhaus M., Tamborrini G., Chary-Valckenaere I., Terslev L., Iagnocco A. (2019). Video clip assessment of a salivary gland ultrasound scoring system in Sjogren′s syndrome using consensual definitions: An OMERACT ultrasound working group reliability exercise. Ann. Rheum. Dis..

[22] Carsons S.E., Vivino F.B., Parke A., Carteron N., Sankar V., Brasington R., Brennan M.T., Ehlers W., Fox R., Scofield H. (2017). Treatment Guidelines for Rheumatologic Manifestations of Sjögren′s Syndrome: Use of Biologic Agents, Management of Fatigue, and Inflammatory Musculoskeletal Pain. Arthritis Care Res..

[23] Vivino F.B., Carsons S.E., Foulks G., Daniels T.E., Parke A., Brennan M.T., Forstot S.L., Scofield R.H., Hammitt K.M. (2016). New Treatment Guidelines for Sjögren′s Disease. Rheum. Dis. Clin. N. Am..

[24] Saraux A., Pers J.O., Devauchelle-Pensec V. (2016). Treatment of primary Sjögren syndrome. Nat. Rev. Rheumatol..

[25] Tomiita M., Kobayashi I., Itoh Y., Inoue Y., Iwata N., Umebayashi H., Okamoto N., Nonaka Y., Hara R., Mori M. (2021). Clinical practice guidance for Sjögren′s syndrome in pediatric patients (2018)—Summarised and Updated. Mod. Rheumatol..

[26] Sumida T., Azuma N., Moriyama M., Takahashi H., Asashima H., Honda F., Abe S., Ono Y., Hirota T., Hirata S. (2018). Clinical practice guideline for Sjogren′s syndrome 2017. Mod. Rheumatol. Jpn. Rheum. Assoc..

[27] Liu L., Tang L., Zhang L., Li X., Huang P., Xiong J., Xiao Y., Liu L. (2022). The first case report of preschool-onset SS/SLE coexisting with NMOSD of Chinese origin. Front. Immunol..

[28] Tarvin S.E., O′Neil K.M. (2018). Systemic Lupus Erythematosus, Sjögren Syndrome, and Mixed Connective Tissue Disease in Children and Adolescents. Pediatr. Clin. N. Am..

[29] Willeke P., Schluter B., Becker H., Schotte H., Domschke W., Gaubitz M. (2007). Mycophenolate sodium treatment in patients with primary Sjogren syndrome: A pilot trial. Arthritis Res. Ther..

[30] Lieberman S.M., Lu A., McGill M.M. (2018). Oral lesions as presenting feature of childhood Sjögren syndrome. Int. J. Pediatr. Otorhinolaryngol..

[31] Means C., Aldape M.A., King E. (2017). Pediatric primary Sjögren syndrome presenting with bilateral ranulas: A case report and systematic review of the literature. Int. J. Pediatr. Otorhinolaryngol..

[32] Takagi Y., Katayama I., Eida S., Sasaki M., Shimizu T., Sato S., Hashimoto K., Mori H., Otsuru M., Umeda M. (2023). Three Signs to Help Detect Sjögren′s Syndrome: Incidental Findings on Magnetic Resonance Imaging and Computed Tomography. J. Clin. Med..

[33] Wang C., Simpkin C., Vielkind M., Galambos C., Lin C., Liptzin D.R., Curran M.L. (2021). Childhood-Onset Sjögren Syndrome Presenting as Pulmonary Hemorrhage. Pediatrics.

[34] Chen H.A., Chen C.H., Cheng H.H. (2009). Hemolytic uremic syndrome and pericarditis as early manifestations of primary Sjögren′s syndrome. Clin. Rheumatol..

[35] Duarte F., Oliveira L., Fontes T., Ramos S., Dourado R., Martins D. (2023). Chronic constrictive pericarditis: A rare cardiac involvement in primary Sjögren′s syndrome. BMC Cardiovasc. Disord..

[36] Gyöngyösi M., Pokorny G., Jambrik Z., Kovács L., Kovács A., Makula E., Csanády M. (1996). Cardiac manifestations in primary Sjögren′s syndrome. Ann. Rheum. Dis..

[37] Davidson D.F., Scott J.G. (2012). Detection of creatine kinase macroenzymes. Ann. Clin. Biochem..

[38] Aljuani F., Tournadre A., Cecchetti S., Soubrier M., Dubost J.J. (2015). Macro-creatine kinase: A neglected cause of elevated creatine kinase. Intern. Med. J..

[39] Thatayatikom A., Jun I., Bhattacharyya I., Berg K., Lee Y.J., Kim Y., Adewumi A., Zhang W., Thatayatikom S., Shah A. (2021). The Diagnostic Performance of Early Sjogren′s Syndrome Autoantibodies in Juvenile Sjogren′s Syndrome: The University of Florida Pediatric Cohort Study. Front. Immunol..

[40] Parker M., Zheng Z., Lasarev M.R., Larsen M.C., Loo A.V., Alexandrids R., Newton M.A., Shelef M.A., McCoy S.S. (2024). Novel autoantibodies help diagnose anti-SSA antibody negative Sjögren disease and predict abnormal labial salivary gland pathology. Ann. Rheum. Dis..

[41] Quéré B., Jousse-Joulin S. (2025). Advances in imaging techniques for Sjogren′s disease. Best Pract. Res. Clin. Rheumatol..

[42] Kim S.H., Min H.K., Lee S.H., Lee K.A., Kim H.R. (2022). Ultrasonographic evaluation of lacrimal glands in patients with primary Sjögren′s syndrome. Clin. Exp. Rheumatol..

[43] Kim S.H., Min H.K. (2024). Clinical utility of salivary and lacrimal gland ultrasonography in primary Sjögren′s syndrome. Clin. Exp. Rheumatol..

[44] Singh S., Jasani G., Basu S., Varma D.R. (2024). Radiological Imaging of the Lacrimal Gland in Sjogren′s Syndrome: A Systematic Review and Meta-Analysis. Curr. Eye Res..

[45] Adeline F., Hittinger A., Bolko L., Guettier C., Kone-Paut I., Schvartz A. (2025). Diagnostic value of minor salivary gland biopsy for Sjögren′s syndrome in children: A monocentric retrospective study over 10 years. Jt. Bone Spine.

